# Bioactivity Profiling of In Silico Predicted Linear Toxins from the Ants *Myrmica rubra* and *Myrmica ruginodis*

**DOI:** 10.3390/toxins14120846

**Published:** 2022-12-02

**Authors:** Sabine Hurka, Tim Lüddecke, Anne Paas, Ludwig Dersch, Lennart Schulte, Johanna Eichberg, Kornelia Hardes, Karina Brinkrolf, Andreas Vilcinskas

**Affiliations:** 1 Institute for Insect Biotechnology, Justus Liebig University Giessen, 35392 Giessen, Germany; 2 LOEWE Centre for Translational Biodiversity Genomics (LOEWE-TBG), 60325 Frankfurt, Germany; 3 Department of Bioresources, Fraunhofer Institute for Molecular Biology and Applied Ecology, 35392 Giessen, Germany; 4 BMBF Junior Research Group in Infection Research “ASCRIBE”, 35392 Giessen, Germany; 5 Bioinformatics and Systems Biology, Justus Liebig University Giessen, 35392 Giessen, Germany

**Keywords:** ant, venomics, biodiscovery, toxin, antibiotics, drug leads

## Abstract

The venoms of ants (Formicidae) are a promising source of novel bioactive molecules with potential for clinical and agricultural applications. However, despite the rich diversity of ant species, only a fraction of this vast resource has been thoroughly examined in bioprospecting programs. Previous studies focusing on the venom of Central European ants (subfamily Myrmicinae) identified a number of short linear decapeptides and nonapeptides resembling antimicrobial peptides (AMPs). Here, we describe the in silico approach and bioactivity profiling of 10 novel AMP-like peptides from the fellow Central European myrmicine ants *Myrmica rubra* and *Myrmica ruginodis*. Using the sequences of known ant venom peptides as queries, we screened the venom gland transcriptomes of both species. We found transcripts of nine novel decapeptides and one novel nonapeptide. The corresponding peptides were synthesized for bioactivity profiling in a broad panel of assays consisting of tests for cytotoxicity as well as antiviral, insecticidal, and antimicrobial activity. U-MYRTX-Mrug5a showed moderately potent antimicrobial effects against several bacteria, including clinically relevant pathogens such as *Listeria monocytogenes* and *Staphylococcus epidermidis*, but high concentrations showed negligible cytotoxicity. U-MYRTX-Mrug5a is, therefore, a probable lead for the development of novel peptide-based antibiotics.

## 1. Introduction

There are more than 14,000 known species of ants (Formicidae) [1,2], which are grouped with bees, wasps, and hornets in the order Hymenoptera [3]. Like many hymenopterans, most ants carry a functional ovipositor-derived venom system that is used primarily for defense and hunting, although it has been reduced to an acid-spraying system in some non-stinging ants. Ant venoms are surprisingly potent, and several ant species cause severe pain or anaphylactic shock even in large victims, including humans [4,5,6,7,8,9].

Ant venoms are complex mixtures of proteins, peptides, and small molecules [5,6]. Some stinging ants (e.g., the genus *Solenopsis*) are known for their alkaloid-dominated venom profile, whereas others (e.g., the genus *Myrmecia*) are known for their venom polypeptide toxins that trigger nociceptive reactions [4,10]. However, ant venoms across the entire family feature relatively short, linear venom peptides [5,6]. These are potent molecules with a range of activities, including the ability to inhibit microbes, resulting in their classification as antimicrobial peptides (AMPs). Some of these AMP-like toxins from ants have been investigated in detail, including ponericins (from *Neoponera goeldii*, *N. apicalis*, and *N. inversa*), dinoponeratoxins (from *Dinoponera australis* and *D. quadriceps*), and bicarinalins (from *Tetramorium bicarinatum*) [5,6,11,12,13,14,15,16,17,18,19]. The biological function of these peptides is generally to facilitate hunting and/or defense [20]. However, the antimicrobial activity of linear AMP-like ant venom peptides may also help to disinfect overpowered prey before they are brought into the colony [21]. This prevents the transfer of pathogens into the colony and protects other ants from infection. The anti-infective properties of crude ant venom and ant venom components could be exploited for the development of new drugs, especially antibiotics [13,22,23,24].

Despite their biological importance and promising translational potential, most ant venoms have not been characterized in any detail. In the past, this reflected the inability of researchers to identify venom components due to the minuscule venom yield of ants and other small arthropods [25,26]. Several milligrams of crude venom are needed as starting material for traditional pharmacology and biofractionation studies, requiring the collection of hundreds or even thousands of specimens [25,26]. More recently, the emerging field of modern venomics has addressed this issue by applying cutting-edge omics technologies to venom systems (particularly transcriptomics and proteomics), making even the smallest venomous animals accessible to investigation [27]. This approach can be combined with chemical synthesis, heterologous expression, or cell-free expression to produce venom components in the laboratory, bringing all venomous species within the reach of basic and translational research programs [27].

We recently investigated the venom systems of two ants from the subfamily Myrmicinae (*Myrmica rubra* and *Myrmica ruginodis*) using a workflow that combined transcriptomics and bottom-up proteomics [28]. The venoms are mixtures of enzymes (e.g., phospholipase A2, serine proteases, and CAP proteins) and potentially neurotoxic cysteine-rich peptides featuring an epidermal growth factor (EGF)-like motif [4]. Interestingly, our study did not detect any short AMP-like toxins, which are common components in many ant venoms. However, at least one such peptide has previously been identified in *M. rubra* and another in its close relative, *Manica rubida* [29,30]. It is, therefore, likely that the venoms of *M. rubra* and *M. ruginodis* contain more AMP-like toxins that were overlooked in a previous study due to their small size, making them difficult to detect using a bottom-up proteomics strategy. We therefore re-examined the venom glands of both species, searching for evidence of AMP-like toxins. Ten novel peptides were identified in silico, and we established their physicochemical properties, likely biological functions, and potential for medical applications by conducting bioactivity profiling using a broad range of assays.

## 2. Results

### 2.1. AMP-like Toxins Are Expressed in M. rubra and M. ruginodis Venom Glands

To determine whether *M. rubra* and *M. ruginodis* venoms contain hitherto unknown short AMP-like toxins, we used the known *M. rubida* peptide U_12_-MYRTX-Mri1a and the known *M. rubra* peptide U_1_-MYRTX-Mr1a as BLAST queries to screen the *M. rubra* and *M. ruginodis* venom gland transcriptomes [28]. We recovered nine additional unique transcripts with high similarity to the query sequences, six in *M. ruginodis* and three in *M. rubra*. We also recovered two additional transcripts encoding identical mature toxins present in both species. The first set of transcripts encodes the toxins named U-MYRTX-Mrub4b or U-MYRTX-Mrug3a depending on its source; therefore, we refer to the sequence hereafter as U-MYRTX-Mrub4b/Mrug3a. The other set encodes U-MYRTX-Mrub4a and U-MYRTX-Mrug7a and is, thus, referred to as U-MYRTX-Mrub4a/Mrug7a. We also found that the *M. rubra* peptide U-MYRTX-Mrub2a was identical to the *M. rubida* query sequence U_12_-MYRTX-Mri1a. Among the 10 peptides we identified, U-MYRTX-Mrug5b was a nonapeptide and the rest were decapeptides.

SignalP revealed that all 10 peptides feature N-terminal signal peptides and are, therefore, likely to be secreted. We identified six principal structural types based on the N-terminal amino acids. The first and most diverse group (the IDP type) features an N-terminal IDP motif and comprises U-MYRTX-Mrub2a, U-MYRTX-Mrug2a, and U-MYRTX-Mrug2b. Our query sequences U_12_-MYRTX-Mri1a and U_1_-MYRTX-Mr1a also carry this motif and were, thus, assigned to the same group. The other toxins were assigned to the IDS group (U-MYRTX-Mrub4a/Mrug7a and U-MYRTX-Mrub4b/Mrug3a), INP group (U-MYRTX-Mrug5a and U-MYRTX-Mrug5b), IDR group (U-MYRTX-Mrub3a), IDV group (U-MYRTX-Mrug4b), and KDS group (U-MYRTX-Mrug6a). These data are summarized in Table 1.

**Figure 1:**
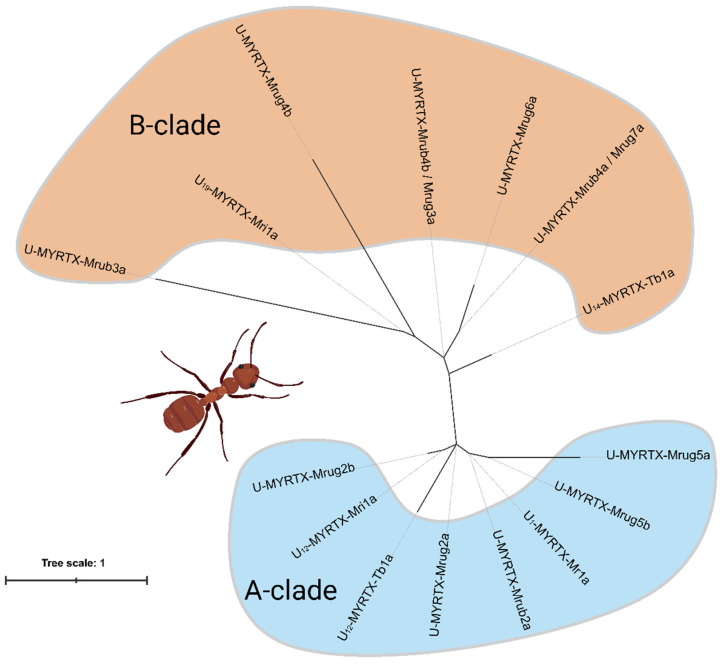
Unrooted phylogenetic tree based on the maximum-likelihood analysis of AMP-like toxins from Central European myrmicine ants (*Myrmica rubra*, *Myrmica ruginodis* and *Manica rubida*, tribus Myrmicini), and a tropical distantly related species (*Tetramorium bicarinatum*, tribus Crematogastrini). The sequences formed two distinct clades. The more diverse A-clade (orange) consists of the structural classes IDP and INP. The less diverse B-clade (blue) consists of the structural classes IDR, IDS, IDV, IPP, LSP, and KDS. The phylogeny is based on amino-acid sequence data from mature ant venom peptides.

Next, we investigated the evolutionary relationships between known myrmicine AMP-like toxins and our novel candidates by phylogenetic analysis (Figure 1). Therefore, we added the mature sequence of U_19_-MYRTX-Mri1a (IDSAAIATLQGGTV) from *M. rubida*, plus the sequences U_12_-MYRTX-Tb1a (LSPAVLASLA) and U_14_-MYRTX-Tb1a (IPPNAVKSLQ) from the more distantly related myrmicine *Tetramorium bicarinatum* to our dataset [31,32]. Similarly to the other toxins assigned to structural classes based on their N-terminal sequence motif, these additional sequences could be subcharacterized. While U_19_-MYRTX-Mri1a displays the IDS motif that we also detected in *Myrmica* toxins, the *Tetramorium* peptides featured two novel N-terminal motifs (LSP in U_12_-MYRTX-Tb1a and IPP in U_14_-MYRTX-Tb1a). Across our phylogeny, the myrmicine toxins formed two major monophyletic clades. The more diverse clade A contained the IDP and INP peptides, while the less diverse B-clade contained the IDR, IDS, IDV, IPP, LSP, and KDS peptides. The distinct structural types did not form monophyletic groups.

### 2.2. No Cytotoxicity but Partly Insecticidal Activity of Tested AMP-like Toxins

**Figure 2:**
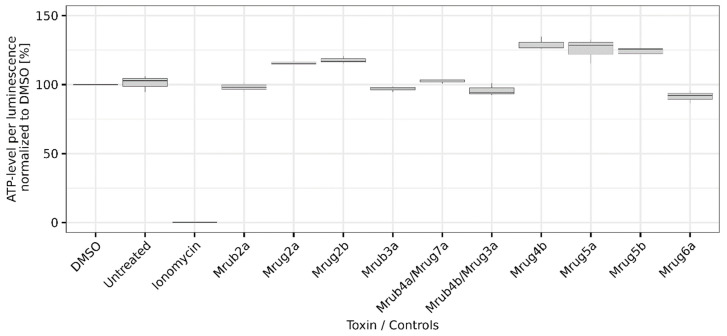
Cytotoxicity of linear venom peptides (100 µM in the assay) from the ants *M. rubra* and *M. ruginodis*. Cytotoxicity was measured using the CellTiter-Glo assay to determine the viability of MDCK II cells. The cytotoxicity of the 10 novel peptides (identified by their short names) was determined in triplicate and compared with untreated cells and cells treated with DMSO and ionomycin as controls. Values were normalized to the DMSO control. Boxplots indicate 0.25, 0.5, and 0.75 percentiles with 1.5 interquartile range.

AMP-like linear ant venom peptides often interact with lipid bilayers in biological membranes. This results in the formation of cytolytic pores via three main mechanisms. To determine whether the *M. rubra* and *M. ruginodis* AMP-like toxins possess cytolytic activity, we exposed canine kidney MDCK II cells to synthetic versions of all 10 peptides. Cytotoxicity was evaluated by quantification of intracellular ATP using the CellTiter-Glo assay, in which luminescence provides an indication of cell viability. The negative control (DMSO) and untreated cells showed no loss of viability. Ionomycin was used as a negative control and showed high cytotoxicity. None of the tested peptides reduced cell viability, confirming their lack of cytotoxicity (Figure 2).

**Figure 3:**
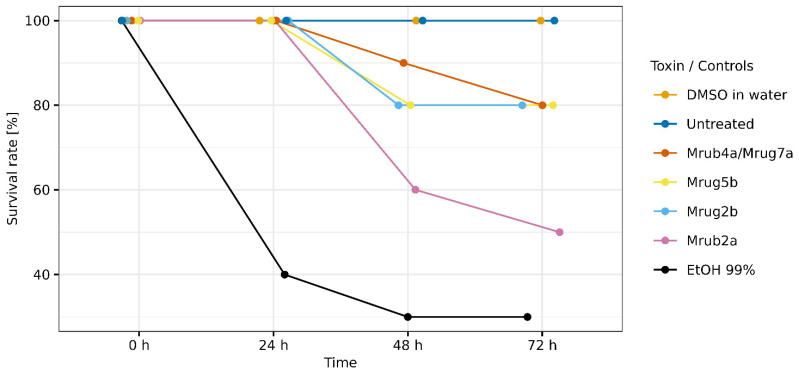
Insecticidal activity of linear venom peptides from the ants *M. rubra* and *M. ruginodis*. Insecticidal activity was determined by injection into larvae of the greater wax moth (*G. mellonella*). Untreated larvae, as well as those injected with 1:1 50% DMSO in water and 99% ethanol, were used as controls. Injected larvae were monitored for 72 h. Peptides are identified by their short names. The position_jitter function of ggplot2 was used to visualize overlapping data points.

We next assessed the insecticidal activity of the toxins using an injection-based assay in larvae of the greater wax moth (*Galleria mellonella*). Injection was deemed a suitable administration route because ants inject their toxins using their modified ovipositor. Four of the 10 peptides showed some degree of insecticidal activity. The effect of U-MYRTX-Mrub4a/Mrug7a, U-MYRTX-Mrug5b, and U-MYRTX-Mrug2b was weak, with 80% of the injected larvae surviving for at least 72 h. In contrast, U-MYRTX-Mrub2a killed 50% of the larvae during the same period (Figure 3). Injection of ethanol (99% purity) caused 70% mortality while the untreated and the 50% DMSO in water (*v*/*v*)-injected specimens did not die.

### 2.3. AMP-like Ant Toxins Exert No Protective Effect against Influenza Virus

**Figure 4:**
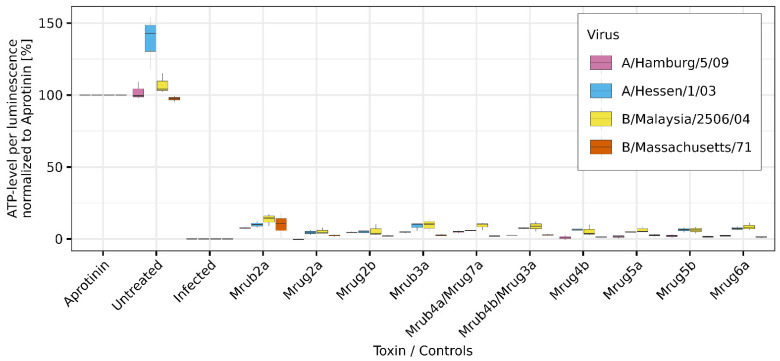
Antiviral screening of linear venom peptides (100 µM in the assay) from the ants *M. rubra* and *M. ruginodis* against four strains of influenza virus in MDCK II cells using the CellTiter-Glo assay. We used the strains A/Hamburg/5/09 (H1N1), A/Hessen/1/03 (H3N2), B/Malaysia/2506/04 (Victoria lineage), and B/Massachusetts/71 (Yamagata lineage). Infected, untreated cells were used as negative controls (no protective effect), and uninfected cells, as well as cells treated with aprotinin, were used as positive controls (100% protection). The peptides are identified by their short names. All measurements were performed as triplicates. Values were normalized to the aprotinin control. Boxplots indicate 0.25, 0.5, and 0.75 percentiles with 1.5 interquartile range.

Some AMP-like toxins from arthropod venoms can inhibit viral infection [33]. We, therefore, tested the *M. rubra* and *M. ruginodis* peptides against four strains of influenza viruses: the influenza A viruses A/Hamburg/5/09 (H1N1) and A/Hessen/1/03 (H3N2), and the influenza B viruses B/Malaysia/2506/04 (Victoria lineage) and B/Massachusetts/71 (Yamagata lineage). The protective effects were determined using the CellTiter-Glo assay, in this case by evaluating the ability of peptides to prevent the infection-induced cytopathic effect in the presence of each virus. However, none of the peptides showed a protective effect against any of the viral strains (Figure 4).

### 2.4. Tested Peptides Display Broad-Spectrum Antimicrobial Effects

**Figure 5:**
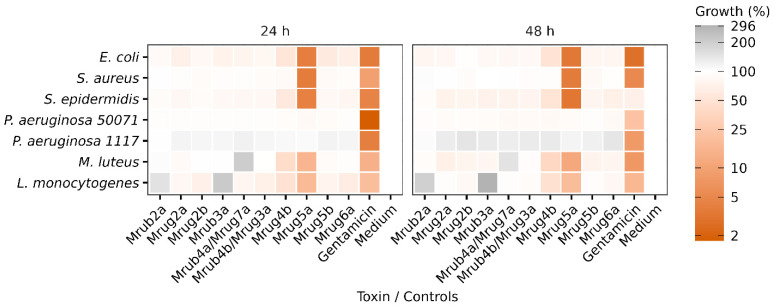
Heat map representing the antibacterial activity of linear venom peptides from the ants *M. rubra* and *M. ruginodis* against seven strains of bacteria. The OD_600_ of triplicate cultures was measured after 24 and 48 h and was normalized to the untreated bacterial controls (set as 100% growth and shown in white). Darker red colors indicate more potent growth inhibition. The antibiotic gentamicin was used as a positive control. The peptides are identified by their short names.

Lastly, we tested the 10 peptides for their activity against a panel of environmentally and/or clinically relevant bacteria: *Escherichia coli*, *Staphylococcus aureus*, *Staphylococcus epidermidis*, *Pseudomonas aeruginosa* (strains 50071 and 1117), *Micrococcus luteus*, and *Listeria monocytogenes*. After 48 h, most of the peptides (U-MYRTX-Mrub2a, U-MYRTX-Mrub4a/Mrug7a, U-MYRTX-Mrub3a, U-MYRTX-Mrub4b/Mrug3a, U-MYRTX-Mrug6a, U-MYRTX-Mrug5b, U-MYRTX-Mrug2a, and U-MYRTX-Mrug2b) showed no significant inhibition of bacterial growth. However, U-MYRTX-Mrug4b inhibited the growth of *E. coli* by 50%, *S. epidermidis* by 48%, *M. luteus* by 62%, and *L. monocytogenes* by 52%. Furthermore, U-MYRTX-Mrug5a substantially inhibited the growth of several bacteria with an efficacy similar to or better than the gentamicin control. Only the two strains of *P. aeruginosa* showed normal growth in the presence of this peptide. After 48 h, this peptide inhibited the growth of *E. coli* by 97%, *S. aureus* by 96%, *S. epidermidis* by 97%, *M. luteus* by 89%, and *L. monocytogenes* by 81%. These results are summarized in Figure 5.

In initial experiments, we used 100 µM of each peptide to ensure unambiguous results, but this does not provide detailed information about the potency of the effects. Given the promising activity profile of U-MYRTX-Mrug5a, we tested this peptide at lower concentrations to determine its potency, and to establish whether its effect is bacteriostatic or bactericidal. The peptide was tested as a dilution series to determine the minimal inhibitory concentration (MIC) and minimum bactericidal concentration (MBC) using 95% inhibition as a cutoff. For *L. monocytogenes*, the MIC and MBC values were both 50 µM. The equivalent values for *M. luteus* were both 6.25 µM, and, for *S. epidermidis* and *E. coli*, they were both 50 µM. The two strains of *P. aeruginosa* remained unaffected by U-MYRTX-Mrug5a; for *S. aureus*, the MIC was 100 µM but the MBC could not be determined. U-MYRTX-Mrug5a, therefore, displays a broad spectrum of activity and shows moderately potent effects against several bacterial strains (Table 2).

## 3. Discussion

Ants are among the most successful groups of terrestrial venomous animals [5,6]. Their venoms offer a rich source of novel bioactive molecules with potential applications in biomedicine and agriculture [13,22,23,24,29,30]. However, the venoms of most ants and other small insects have not been characterized, even at a superficial level [5,6,34]. This is particularly true for members of the subfamily Myrmicinae, the dominant ant lineage in the temperate parts of Central Europe [1].

A few members of this subfamily have been studied using modern methods to determine their venom components. Examples include *M. rubra* and *M. ruginodis*, two myrmicine ants that are widely distributed in Europe. Their venom was studied by proteo-transcriptomics and was found to contain several potential neurotoxins with an EGF fold, as well as many enzymes [28]. However, a bottom-up proteomics strategy was used in the original study, in which the crude venom is digested with trypsin before LC–MS analysis. This method is ideal for the detection of polypeptides >20 amino acids in length, but often fails to identify components that are significantly shorter. Therefore, although linear AMP-like toxins are widespread venom components in ants [5,6], their presence was not detected in the original study [28]. This is underlined by recent studies using a different MS-based approach, which identified and tested several AMP-like toxins from *M. rubra* and *M. rubida* [29,30]. We, therefore, screened the previously published transcriptome datasets for transcripts encoding putative AMP-like toxins. Our in silico search of venom gland transcriptomes identified 10 transcripts resembling known ant AMP-like toxins, showing that the venom glands of *M. rubra* and *M. ruginodis* do indeed express such components. This does not prove that the transcripts are translated into polypeptides, and this must be confirmed in future studies. However, the expression of such peptides is likely, given that similar peptides have already been detected in *M. rubra* and *M. rubida* [29,30]. This also underlines the need to combine different venomics technologies in order to provide a holistic picture of the chemical profile in a given venom system. The combination of transcriptomics with top-down proteomics and/or peptidomics is a particularly desirable addition to bottom-up proteomics because these methods can identify even the smallest polypeptide toxins. Moreover, in the present study we focused on the prediction of AMP-like toxins with similarity to two known ant toxins of this type. However, several additional myrmicine AMP-like toxins have been described including U_19_-MYRTX-Mri1a, U_12_-MYRTX-Tb1a, and U_14_-MYRTX-Tb1a. Nevertheless, these are either of different length (>10 amino acids) than our herein employed query sequences or stem from relatively distantly related species (Crematogastrini). Toxin length is an important factor in translational bioprospecting programs, with shorter toxins being preferably used. Furthermore, phylogenetic distance may have an impact on the accuracy of predictions thanks to different evolutionary forces and selection pressures at play. Therefore, we focused in this work on the deca- and nonapeptides from very closely related species from the same tribe. That said, a broader investigation using additional sequences from other myrmicine tribes, such as Attini or Crematogastrini, will certainly yield additional peptides. Therefore, we recommend that future studies should pursue such a taxonomically inclusive strategy. Nevertheless, purely transcriptome-based approaches tend to overestimate toxin diversity; in particular, de novo transcriptomics can be spurred by erroneous assemblies. For this reason, predicted sequences may not necessarily represent the true mature peptide, and mass spectrometry data should be used to support such future studies if possible.

Ants use venom primarily to defend their colony against threats and secondarily to overpower prey [20]. The role of AMP-like toxins in these defensive and trophic scenarios is unclear. We tested the activity of our peptides in a range of assays that could provide insights into a potential defensive function. However, they showed no toxicity toward MDCK II cells, suggesting that a defensive function against mammalian predators may be ruled out. They also showed only marginal insecticidal activity against greater wax moth larvae, with 24–48 h between injection and death. There were no immediate effects, which would typically be required in a defensive scenario to prevent further attack against the defended colony. A similar rapid onset of intoxication symptoms is required in trophic scenarios because prey that cannot be subdued immediately is more likely to escape. Although some known AMP-like toxins show insecticidal activity [21,29,30,35], they are unlikely to function as trophic weapons given the slow onset of lethal effects.

When ants have overpowered their prey, it is transferred to the colony as a source of nutrition [3]. If contaminated with pathogenic bacteria, the carcass represents a potential source of infection that could cause a massive outbreak of disease. It is, therefore, possible that antimicrobial ant venom components, including AMP-like toxins, play a semitrophic role as disinfectants, primarily to sanitize prey [21,36], in addition to during self-grooming and allogrooming [3,37] or for the sanitation of nest material and brood [38,39]. By co-injecting these components into prey, the prey microbiome could be destroyed and the prey sterilized. This would reduce the likelihood of infections arising from pathogen-rich prey carcasses, leading to a selective advantage in the colony. Our activity screen revealed that myrmicine AMP-like toxins show antimicrobial activity against several bacteria, supporting the proposed semi-trophic role to sterilize prey and to defend the colony against pathogens.

Ant venoms are a rich source of novel bioactive molecules with potential applications in medicine and agriculture [13,22,23,24,29,30]. We evaluated the translational potential of the 10 peptides by testing their activity against viruses, bacterial pathogens, and insect pests. The injection into *G. mellonella* larvae revealed that one of the peptides caused 50% mortality, but this effect is not potent enough to justify further development as a bio-insecticide. There was no activity against four influenza virus strains, indicating that none of the peptides are suitable as antiviral drug candidates. However, several of the peptides showed activity against pathogenic bacteria, and high concentrations (100 μM) of U-MYRTX-Mrug5a outperformed gentamicin, most significantly when tested against *L. monocytogenes*. Subsequent analysis showed that this peptide has mostly bactericidal effects and retains its activity against some bacteria even at lower concentrations. However, the MIC was at least 50 μM against all bacterial targets. The native peptide is, therefore, not outstandingly potent, but its lack of cytotoxicity paired with its broad activity spectrum suggests it is a promising lead for further development. Future studies should investigate whether peptide engineering can increase its potency while maintaining its low cytotoxicity.

## 4. Conclusions

Ants are among the most successful lineages of venomous animals, but remarkably little is known about the components and activity of their venoms, particularly in the subfamily Myrmicinae. We used an in silico approach to identify and predict the primary sequence of 10 novel AMP-like toxins in the venom gland transcriptomes of the myrmicine ants *M. rubra* and *M. ruginodis*. We then synthesized all 10 peptides and assessed their bioactivity profiles using a screening pipeline that included a broad range of assays. The peptides showed no cytotoxicity or antiviral activity, but several of them showed mild effects against greater wax moth larvae. More importantly, many of the peptides showed some form of antimicrobial activity, with U-MYRTX-Mrug5a achieving the most potent bactericidal effects. We hypothesize that these toxins are not used as defensive weapons but may facilitate predator–prey interactions by helping to subdue prey and/or by sterilizing the prey before it is transferred to the colony. The bioactivity profile of U-MYRTX-Mrug5a combines low cytotoxicity with activity against clinically relevant bacteria, suggesting it could be developed as a novel antibiotic. The native peptide probably lacks sufficient potency, but peptide engineering could overcome this limitation. Our data confirm that novel bioactive molecules with translational potential can be identified in myrmicine ant venoms, highlighting the importance of neglected ants for future venom bioprospecting programs.

## 5. Material and Methods

### 5.1. Identification of Peptides

AMP-like peptides in *M. rubra* and *M. ruginodis* were identified by screening the recently published venom gland transcriptome data for both species [28] using BLASTP v2.11.0 [40] with the following settings: -evalue 10 -word_size 3 -matrix BLOSUM62 -max_target_seqs 500 -seg ‘no’. We used two known AMP-like peptide sequences as queries (*M. rubida* U_12_-MYRTX-Mri1a [30] and *M. rubra* U_1_-MYRTX-Mr1a [29]) and matched them against predicted open reading frames generated from the transcriptome assemblies using TransDecoder v5.5.0 [41] as previously described [28]. Only candidates with a signal peptide predicted using SignalP v6.0g [42] in slow sequential mode were retained. We aligned the sequences using MAFFT v7.496 [43] in L-INS-I mode and used SignalP results and information from UniProtKB 2022_02 [44] to manually inspect the alignments and to select candidate peptides. We presumed a maximum of 10 amino acids per candidate peptide which is not necessarily the full mature peptide. The in vivo bioprocessed peptides may vary from our assumption. Full BLAST results and alignments are available in Supplementary Data S1–S3.

### 5.2. Phylogenetic Analysis

The mature amino-acid sequences of the 10 novel peptides, the query sequences U_12_-MYRTX-Mri1a and U_1_-MYRTX-Mr1a, and the additional sequences U_19_-MYRTX-Mri1a, U_14_-MYRTX-Tb1a and U_12_-MYRTX-Tb1a were aligned using the online version of MAFFT v7.505 with default settings. A maximum likelihood phylogenetic tree was then built using IQtree v1.6.12 [45] with the following settings: sequence type = protein, substitution model = auto, bootstrap analysis = ultrafast, number of bootstrap alignments = 1000, maximum iterations = 10,000, minimum correlation coefficient = 0.99, single-branch test replicates = 1000, perturbation strength = 0.5, and IQ tree stopping rule = 100. The resulting consensus tree was visualized in iTOL v6.6 [46]. The evolutionary history of the peptides is unclear and no close relatives are known; hence, we did not add an outgroup and we exclusively calculated unrooted trees. The final consensus tree file is available as Supplementary Data S4.

### 5.3. Peptide Nomenclature

The novel peptides were named as previously described [6], based on a modification of the King nomenclature [47]. All names were given the prefix U to indicate an unknown mechanism of action. The abbreviation MYRTX (myrmicitoxin) was used to indicate their origin in myrmicine ants followed by the species descriptors Mrug (*M. ruginodis*) or Mrub (*M. rubra*).

### 5.4. Peptide Synthesis

The peptides were produced by solid-phase synthesis followed by lyophilization (GenScript Biotech, Rijswijk, the Netherlands). All 10 peptides were synthesized as N-terminal amides because all previously identified AMP-like ant toxins carry this modification.

### 5.5. Cytotoxicity Assays

Madin–Darby canine kidney II (MDCK II) cells kindly provided by Prof. Dr. Friebertshäuser (Philipps University Marburg, Institute of Virology, Marburg, Germany) were maintained in Dulbecco’s modified Eagle’s medium (DMEM GlutaMAX) supplemented with 1% penicillin/streptomycin and 10% fetal calf serum (all reagents from Thermo Fisher Scientific, Walthman, MA, USA) and were grown at 37 °C in a 5% CO_2_ atmosphere. The peptides or ionomycin (Cayman Chemical, Ann Arbor, MI, USA) as a positive control were dissolved in DMSO to prepare 10 mM stock solutions, which were stored at −20 °C. MDCK II cells were seeded in a 96-multiwell plate and grown to 90% confluence before treatment with 100 µM of the peptides with a final concentration of 1% DMSO or ionomycin, or with DMSO as a negative control. After incubation for 48 h as above, cell viability was determined using the CellTiter-Glo kit (Promega, Walldorf, Germany). Luminescence was measured in black 96-multiwell plates using a Synergy H4 microplate reader (BioTek/Agilent Technologies, Waldbronn, Germany). Relative light units (RLU) were normalized to the DMSO control set to 100% viability. Triplicate measurements were used to calculate means and standard deviations. Raw data are available in Supplementary Data S5.

### 5.6. Antiviral Assay

Influenza virus strains A/Hamburg/05/2009 (H1N1), A/Hessen/1/2003 (H3N2), B/Malaysia/2506/2004 (B/Mal), and B/Massachusetts (B/Mass) were kindly provided by Prof. Dr. Friebertshäuser (Philipps University Marburg, Institute of Virology, Marburg, Germany) and were propagated in MDCK II cells in infection medium (DMEM GlutaMAX supplemented with 1% penicillin/streptomycin, 0.2% bovine serum albumin (BSA), and 1 µg/mL TPCK-treated trypsin; all reagents from Thermo Fisher Scientific) using a multiplicity of infection (MOI) between 1 and 0.0001. For antiviral screening, MDCK II cells were grown to 90% confluence and inoculated at MOI = 1 for H1N1, H3N2, and B/Mal or at MOI = 0.01 for B/Mass in infection medium without trypsin. After 1 h, the cells were washed twice with PBS, followed by treatment with 100 µM peptides with a final concentration of 1% DMSO or aprotinin (Carl Roth, Karlsruhe, Germany) in infection medium containing 1 µg/mL TPCK-treated trypsin. Cell viability was determined 48 h after treatment using the CellTiter-Glo kit as described above. All values were normalized to the aprotinin treatment (100% viability). Triplicate measurements were used to calculate means and standard deviations. Raw data are available in Supplementary Data S5.

### 5.7. Insecticidal Activity

Greater wax moth larvae were obtained from Fauna Topics Zoobedarf Zucht und Handels GmbH (Marbach am Neckar, Germany). Larvae at development stages L5/L6 (weight of about 500 mg), those with wounds, and those with low vitality were discarded. The remaining larvae were assigned to groups of five and were placed in vented Petri dishes (94 × 16 mm) lined with paper towel to absorb feces. The Petri dishes were placed on ice 5 min before injection to immobilize the insects. The peptides were dissolved in DMSO and mixed with an equal volume of deionized water to make 10 mM stocks immediately before injection. The stock solutions were drawn into an Omnican-F (0.30 × 12 mm/G 30 × 1/2”) 1 mL syringe and adjusted for a World Precision Instruments manual microsyringe injector. We then injected 5 µL of each solution, corresponding to 50 ng of the peptide and 2.5 µL of DMSO, into the pseudopodium of the immobilized larva between two segmental plates, facilitating the spread of the injected components. Water and DMSO/water were used as negative controls (alongside untreated specimens), and 10 µL of 90% ethanol was used as a positive control. All peptide and control groups were tested in 10 specimens. After injection, the larvae were maintained in a Bindner KBWF 240 climate chamber at room temperature in the dark and monitored for survival every 24 h for 3 days.

### 5.8. Antimicrobial Activity

Cryo-conserved cultures (Table 3) were transferred with a sterile inoculation loop to tryptic soy agar (TSA) plates (Carl Roth) sealed with Parafilm (BEMIS, Neenah, WI, USA), and were incubated for 1–2 days at 37 °C depending on growth rates. Plates were then stored at 4 °C. For each of the three replicates, single colonies were picked with a sterile pipette tip, transferred into 2–3 mL Mueller-Hinton II (MH II) medium (BD, Heidelberg, Germany) and cultivated for ~24 h in unsealed 15 mL reaction tubes at 37 °C, shaking at 180 rpm. We then diluted 30 µL (60 µL for *M. luteus* and *L. monocytogenes*) of the bacterial suspension in 2–3 mL of MH II medium in unsealed 15 mL reaction tubes and incubated them as above for 2–3 h. We measured the optical density at 600 nm (OD_600_) by transferring 1 mL of bacterial suspension to a polystyrene cuvette (SARSTED, Nümbrecht, Germany) in an Ultrospec 10 photometer (Biochrome, Cambridge, UK). We then diluted the cultures with MH II medium to the preferred OD_600_ (Table 3) in 2 mL reaction tubes if necessary. The peptides were dissolved in DMSO as described above and diluted with MH II medium to a concentration of 200 µM before the experiments. We used gentamicin (Sigma-Aldrich, Taufkirchen, Germany) as a positive control, generally at a concentration of 10 µM, but at 60 µM for *S. epidermidis*.

The experiments were carried out in 96-multiwell plates with a final volume of 100 µL per well (50 µL of bacterial suspension; 50 µL of testing peptide with a final concentration of 1.9% DMSO). Duplicate plates were sealed carefully with Parafilm, placed in a loosely closed plastic bag, and incubated for 48 h at 37 °C, shaking at 180 rpm. The OD_600_ was measured after 0, 24, and 48 h using a BioTek Eon microplate reader and Gen5 v2.09. One experiment was carried out with automatic OD_600_ measurement every 20 min with a prior 30 s double orbital rotation at 237 rpm with overall incubation at 37 °C for 60 h in the microplate reader.

The MIC for U-MYRTX-Mrug5a was determined by testing nine log_2_ dilutions of all strains in triplicate using the same procedure described above (including one replicate in the microplate reader). For all 96-multiwell plate experiments, we used the following controls: MH II medium instead of peptides with each bacterial strain, medium only with each peptide, and one well with 100 µL of medium only. The MBC was determined by carefully transferring 20 µL per dilution from the 48 h MIC experiments (n = 2) to sectored dry TSA plates as previously described [48]. For each replicate, the OD_600_ value of the medium was used as a blank. The absolute value of the lowest resulting negative value was added to gain only positive values. Wells with medium plus bacteria were set as 100% growth, and the corresponding growth rates in the peptide-treated cultures were calculated in proportion. We used Microsoft Excel and R v4.1.3 [49] with packages plater v1.0.4 [50], tidyverse v1.3.1 [51], and reshape2 v1.4.4 [52] to analyze and visualize the data. Heat maps were generated using a log_10_ transformed scale for better visualization.

To determine the number of colony-forming units (CFUs), we prepared 1:10 serial dilutions of the bacterial suspensions starting with the OD_600_ values in Table 3. The dilutions were prepared in MH II medium, and the cultures were vortexed between steps. We transferred 20 µL of each dilution onto sectored dry TSA plates as previously described [48] with three replicates per strain and dilution. After incubation for 24 h at 37 °C (both *P. aeruginosa* strains also at 30 °C), the colonies were counted. We used the highest countable concentration to calculate the mean CFU/mL for each bacterial strain. Raw data are available in Supplementary Data S6 and S7. A translation from contig to internal identifiers to toxin names is available in Supplementary Data S8.

## Figures and Tables

**Table 1:** AMP-like toxins identified in the *M. rubra* and *M. ruginodis* transcriptomes, as well as the query peptides from *M. rubra* and *M. rubida* (marked with asterisks). The peptides are categorized by formal name, source organism, sequence, structural class, and number of amino acids (#AA).

Toxin	Species	Sequence	Class	#AA
U_12_-MYRTX-Mri1a *	*M. rubida*	IDPKLLESLA	IDP	10
U_1_-MYRTX-Mr1a *	*M. rubra*	IDPKVLESLV	IDP	10
U-MYRTX-Mrub2a	*M. rubra*	IDPKLLESLA	IDP	10
U-MYRTX-Mrug2a	*M. ruginodis*	IDPKVLESLA	IDP	10
U-MYRTX-Mrug2b	*M. ruginodis*	IDPKVLESLL	IDP	10
U-MYRTX-Mrub3a	*M. rubra*	IDRSEKTERE	IDR	10
U-MYRTX-Mrub4a/Mrug7a	*M. rubra* and *M. ruginodis*	IDSDALKSLQ	IDS	10
U-MYRTX-Mrub4b/Mrug3a	*M. rubra* and *M. ruginodis*	IDSKAIKSLQ	IDS	10
U-MYRTX-Mrug4b	*M. ruginodis*	IDVYFILHLP	IDV	10
U-MYRTX-Mrug5a	*M. ruginodis*	INPKLWLKLF	INP	10
U-MYRTX-Mrug5b	*M. ruginodis*	INPKLLESL	INP	9
U-MYRTX-Mrug6a	*M. ruginodis*	KDSDSLKSFQ	KDS	10

**Table 2:** Minimum inhibitory concentration (MIC) and minimum bactericidal concentration (MBC) of peptide U-MYRTX-Mrug5a against various strains of bacteria. The concentration required to inhibit at least 95% of growth is compared to the control. The MIC for *L. monocytogenes* was determined manually from the MBC test plus information from the MIC growth curve.

Strain	MIC [µM]	MBC [µM]
*Listeria monocytogenes*	(50)	50
*Micrococcus luteus*	6.25	6.25
*Pseudomonas aeruginosa* 50071	None	None
*Pseudomonas aeruginosa* 1117	None	None
*Staphylococcus aureus*	100	not to determine
*Staphylococcus epidermidis*	50	50
*Escherichia coli* DE3	50	50

**Table 3:** Bacterial strains used to determine the antimicrobial activity of ant venom peptides. The strains are identified by name, unique identifier, starting OD_600_ value, and corresponding CFU/mL.

Name	Unique Identifier	OD_600_ for Assay	CFU/mL
*Listeria monocytogenes*	DSM 20600	0.000625	1.45 × 10⁶
*Micrococcus luteus*	DSM 20030	0.005	2.20 × 10⁵
*Pseudomonas aeruginosa* 50071	DSM 50071	0.00125	4.83 × 10^8^
*Pseudomonas aeruginosa* 1117	DSM 1117	0.005	3.00 × 10^13^
*Staphylococcus aureus*	DSM 2569	0.00125	9.00 × 10⁶
*Staphylococcus epidermidis*	ATCC 35984; DSM 28319	0.000625	1.73 × 10⁶
*Escherichia coli* DE3	BL21(DE3)	0.000325	2.00 × 10⁶

## Data Availability

Raw transcriptomic data are available at the NCBI database (BioProject PRJNA807911). Measured data can be found in the Supplementary Materials.

## Supplementary Materials

The following supporting information can be downloaded at https://www.mdpi.com/article/10.3390/toxins14120846/s1: Supplementary Data S1 and S2. BLAST results of search in predicted ORF list with U12-MYRTX-Mri1a and U1-MYRTX-Mr1a as query; Supplementary Data S3. Resulting alignment from MAFFT run to validate selected contigs; Supplementary Data S4. The final consensus tree file in NEWICK format; Supplementary Data S5. Raw and normalized data from cytotoxicity and antiviral screening tests; Supplementary Data S6. Raw data of antimicrobial test assays; Supplementary Data S7. Raw data of minimal inhibitory concentration experiments; Supplementary Data S8. Translation from contig to internal identifiers and toxin names.
